# Global migration and factors influencing retention of Asian internationally educated nurses: a systematic review

**DOI:** 10.1186/s12960-024-00900-5

**Published:** 2024-03-01

**Authors:** Danny Shin Kai Ung, Yong Shian Goh, Ryan Yuan Sheng Poon, Yongxing Patrick Lin, Betsy Seah, Violeta Lopez, Kristina Mikkonen, Keng Kwang Yong, Sok Ying Liaw

**Affiliations:** 1 Alice Lee Centre for Nursing Studies, Yong Loo Lin School of Medicine, National University of Singapore, Singapore, Singapore; 2 Coronary Care Unit, Tan Tock Seng Hospital, Singapore, Singapore; 3 Nursing Service, Tan Tock Seng Hospital, Singapore, Singapore; 4 School of Nursing, Midwifery and Social Sciences, Central Queensland University, Rockhampton, Australia; 5 Research Unit of Health Sciences and Technology, University of Oulu, Oulu, Finland; 6 Medical Research Center Oulu, Oulu University Hospital and University of Oulu, Oulu, Finland; 7 National Healthcare Group, Ministry of Health, Singapore, Singapore

**Keywords:** Asian, Migrant, Nurse, Retention, Acculturation

## Abstract

**Background:**

Given nurses’ increasing international mobility, Asian internationally educated nurses (IENs) represent a critical human resource highly sought after within the global healthcare workforce. Developed countries have grown excessively reliant on them, leading to heightened competition among these countries. Hence, this review aims to uncover factors underlying the retention of Asian IENs in host countries to facilitate the development of more effective staff retention strategies.

**Methods:**

A mixed-methods systematic review was conducted using the Joanna Briggs Institute methodology for mixed-method systematic review. A search was undertaken across the following electronic databases for studies published in English during 2013–2022: CINAHL, Embase, PubMed, Scopus, Web of Science and PsycINFO. Two of the researchers critically appraised included articles independently using the Joanna Briggs Critical Appraisal Tools and Mixed Methods Appraisal Tool (version 2018). A data-based convergent integrated approach was adopted for data synthesis.

**Results:**

Of the 27 included articles (19 qualitative and eight quantitative), five each were conducted in Asia (Japan, Taiwan, Singapore and Malaysia), Australia and Europe (Italy, Norway and the United Kingdom); four each in the United States and the Middle East (Saudi Arabia and Kuwait); two in Canada; and one each in New Zealand and South Africa. Five themes emerged from the data synthesis: (1) desire for better career prospects, (2) occupational downward mobility, (3) inequality in career advancement, (4) acculturation and (5) support system.

**Conclusion:**

This systematic review investigated the factors influencing AMN retention and identified several promising retention strategies: granting them permanent residency, ensuring transparency in credentialing assessment, providing equal opportunities for career advancement, instituting induction programmes for newly employed Asian IENs, enabling families to be with them and building workplace social support. Retention strategies that embrace the Asian IENs’ perspectives and experiences are envisioned to ensure a sustainable nursing workforce.

**Supplementary Information:**

The online version contains supplementary material available at 10.1186/s12960-024-00900-5.

## Background

Nurses constitute more than half of the global healthcare workforce and are integral to providing patient treatment and ensuring continuity of care [1]. The World Health Organization has estimated that there is already a shortage of 5.9 million nurses before the 2019 coronavirus disease (COVID-19) pandemic [2]. Amidst this shortage, internationally educated nurses (IENs) have proved to be a critical resource for healthcare systems worldwide. According to the *State of the World’s Nursing Report 2020* by the World Health Organization, nurses’ international mobility is increasing—one in every eight nurses practises in a foreign country [3]. In this regard, Asian IENs, originating mainly from the Philippines and India [4], represent a significant proportion of the migrant healthcare workforce in developed countries, such as Australia [5], New Zealand [6], the United Kingdom [7] and the United States [8], which highlights these countries’ reliance on foreign-trained nurses. Nonetheless, migration is no simple task [9]: Asian IENs must leave their comfort zone and face challenges in immigration, credentialing, and differences in cultures, clinical practices and values [10]. However, such difficulties have not deterred their migration across host countries. A segment of New Zealand-based Asian IENs have been reported to consider nursing prospects abroad for future career development [11]. Asian IENs also migrate within Asian countries before transitioning to countries such as the United States and the United Kingdom. Therefore, such occurrences have presented a situation where some Asian countries, such as Singapore and Malaysia, have become transit country for Asian IENs seeking further migration [11]. In particular, Singapore is a valuable stepping stone for these Asian IENs who migrate to other host countries [12]. This is supported by reports which cited that 14.8% of the Asian IENs in Singapore left the public sector workforce in 2021 [13].

In the era of globalisation, the escalating demand for nurses demonstrated and exacerbated by the COVID-19 pandemic has endowed Asian IENs with greater autonomy in deciding their places of practice. Countries such as Germany, the United Arab Emirates, the United Kingdom and Singapore have introduced various strategies to recruit IENs, such as providing fair wages, free travel, language training, and incentives for passing licensure examinations, easing the recognition of foreign professional qualifications; and expediting visa approvals [14–16]. Thus, these strategies have prompted an influx of IENs from their countries of origin or other developed countries where they work.

While international recruitment may represent a quick-fix option, healthcare systems experiencing excessive out-migration of their IENs should address their retention effectively. This perspective is critical given the substantial costs associated with high turnover [17]. Besides economic effects, rapid staff turnover can cause unfamiliarity among staff members in the interprofessional group and affect interprofessional collaboration, leading to negative patient outcomes. The likely adverse patient outcomes include increased patient falls, incidences of pressure ulcers, the average length of patient stay, and medication errors [18]. Consequently, countries should strategise to ensure the sustained retention of their Asian IENs to mitigate rapid turnover and continue international recruitment.

Many studies have investigated IENs’ lived experiences about aspects such as transition [19], integration [20] and resilience [21]. Although such general experiential insights are valuable, only one review by Pressley et al. [21] has specifically examined the experiences of IENs employed in different countries to provide knowledge on their retention in an overseas position. However, they limited the scope of their review to studies that focused on five developed countries and used qualitative research designs, with most such studies characterising only the initial migratory stages. Thus, there is a gap in knowledge regarding the experiences related to the long-term retention of IENs.

Notably, the experiences of Asian IENs have not been explored in any systematic reviews. Further, despite being the continent with the largest suppliers of nurses, Asia registers the lowest density of nurses worldwide. Many high-income countries rely excessively on IENs due to their inadequate domestic supply or the worsening of their shortages induced by the COVID-19 pandemic [3]. Given the increasing demand for nurses and a depleting supply (suboptimal retention), countries conventionally inactive in international recruitment have begun turning to Asian IENs to fill their vacancies [22]. The resultant escalating competition between countries for IENs, coupled with the ever-growing global shortage of nurses, underscores the urgency to retain the current strength of this workforce. To this end, this systematic review of Asian IENs offers a critical examination of factors influencing their retention in host countries, in line with which the relevant authorities can formulate strategies to ensure a sustainable healthcare workforce.

## Methods

In this systematic review, we adopted the Joanna Briggs Institute (JBI) methodology for mixed-methods systematic reviews through a data-based convergent integrated approach [23].

### Search strategy

**Fig. 1:**
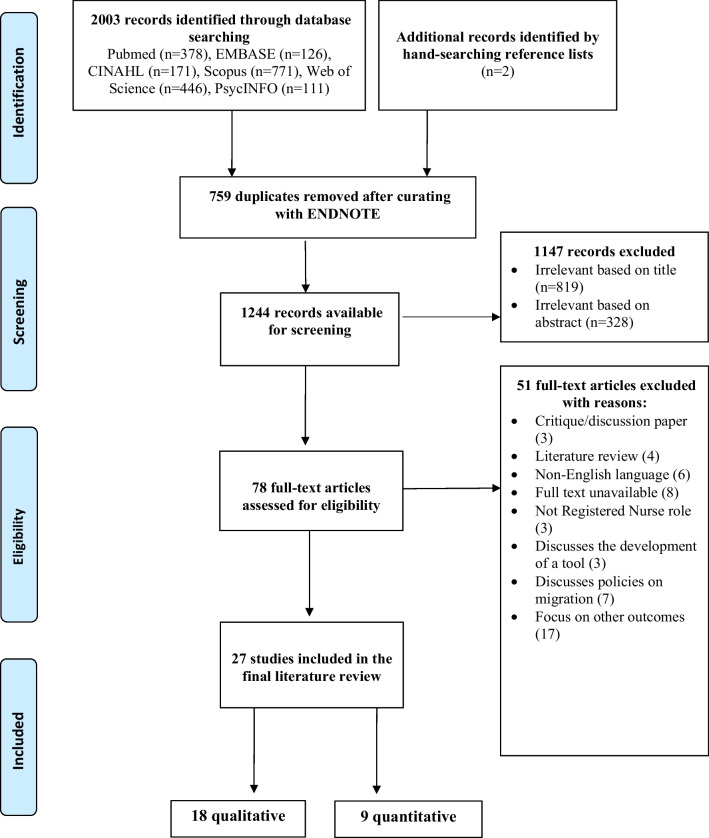
PRISMA flow diagram documenting the search process

A preliminary search was performed on PubMed and Embase to identify relevant articles. Words in the titles and abstracts of identified articles were used to formulate a comprehensive search strategy with an experienced librarian from the Medical Library for guidance. Key search terms included ‘Asian migrant nurses’, ‘retention’ and ‘experience’. Then, a second comprehensive electronic search was performed across CINAHL, Embase, PubMed, Scopus, Web of Science and PsycINFO. The search terms and strategies for each database are provided in Additional file 1. The references of all identified studies were also screened for additional relevant studies. As per the eligibility criteria (see Table 1), studies published in English during 2013–2022 were included. A bibliography management software, EndNote X9 [24], was used to import all the studies and remove duplicates. The titles and abstracts were screened independently by two reviewers (DU and RP) against the inclusion criteria. The selection process is illustrated in a PRISMA flow diagram (Fig. 1).

**Table 1:** Selection criteria for the studies

Criteria	Inclusion	Exclusion
Population	• Registered nurses who received education and attained their nursing qualification prior to being recruited to work in a different country • Migrant nurses of Asian ethnicity. The Asian ethnicities include Chinese, Filipino, Indian, Japanese, Korean, Malaysian, Nepalese, Singaporean, and Taiwanese • Asian migrant nurses practising in inpatient and outpatient clinical settings. This includes acute care, community and primary health care	• Asian migrant nurses make up the minority of the participants • Advanced practice nurses • Physicians • Allied healthcare professionals
Outcomes	• Experiences • Attitudes • Perceptions • Retention • Turnover • Intention to leave • Job satisfaction	
Type of design	• Quantitative studies • Qualitative studies • Mixed methods	
Publication type	Published primary research	• Abstracts only • Reviews • Discussions • Seminar papers or editorials
Language	English	Articles not in English
Year of publication	January 2013 to December 2022	Studies published before January 2013

### Study selection

Eligible articles were retrieved in full and reviewed. Two reviewers (DU and RP) independently used a standardised data-extraction template to extract the following study details: author(s), year of publication, country, study aim(s), methodology, sample characteristics, themes, and primary findings pertinent to the review question. Differences were resolved through discussion with a third reviewer (SYL).

### Assessment of methodological quality

The JBI checklists for analytical cross-sectional studies [25] and for qualitative research [26] were used to appraise quantitative and qualitative studies, respectively [27]. The methodological quality of each study was rated as low (0–49%), medium (50–75%) or high (75–100%). Two reviewers (DU and RP) performed separate critical appraisals. The research team resolved any discrepancies in appraisals through discussions. All studies were included, regardless of their methodological rating (see Tables 2, 3), to ensure that all available evidence could be integrated to maximise the understanding of factors influencing Asian IENs retention.

**Table 2:** JBI critical appraisal checklist for analytical cross-sectional studies

Authors and year	Article title	C1	C2	C3	C4	C5	C6	C7	C8	Score	Rating
An et al. (2014)	Factors Affecting Job Satisfaction of Immigrant Korean Nurses	Y	Y	Y	N/A	Y	Y	Y	Y	7/7	100%
Geun et al. (2016)	Turnover and Associated Factors in Asian Foreign-Educated Nurses	Y	Y	Y	N/A	Y	Y	Y	Y	7/7	100%
Goh & Lopez (2016)	Job satisfaction, work environment and intention to leave among migrant nurses working in a publicly funded tertiary hospital	Y	Y	Y	N/A	Y	Y	Y	Y	7/7	100%
Geun et al. (2018)	Predictors of Turnover Among Asian Foreign-Educated Nurses in Their 1st Year of US Employment	Y	Y	Y	N/A	Y	Y	Y	Y	7/7	100%
Alshareef et al. (2020)	Identifying the factors influencing Saudi Arabian nurses' turnover	Y	Y	Y	N/A	Y	N	Y	Y	6/7	85.71%
Lee et al. (2021)	Job satisfaction of foreign-educated nurses in Malaysia: A cross-sectional study	Y	Y	Y	N/A	N	N	Y	Y	5/7	71.43%
Primeau et al. (2021)	Correlates of career satisfaction in internationally educated nurses: A cross-sectional survey-based study	Y	Y	Y	N/A	Y	Y	Y	Y	7/7	100%
Zanjani et al. (2021)	Overseas qualified nurses’ sociocultural adaptation into the Australian healthcare system: A cross-sectional study	Y	Y	Y	N/A	Y	Y	Y	Y	7/7	100%

**Table 3:** JBI critical appraisal checklist for qualitative research

Authors and year	Article title	C1	C2	C3	C4	C5	C6	C7	C8	C9	C10	Score	Rating
Adhikari and Melia (2013)	The (mis)management of migrant nurses in the UK: a sociological study	Y	Y	Y	Y	Y	Y	N	N	N	Y	7/10	70%
Zhou et al. (2014)	The Experience of China-Educated Nurses Working in Australia: A Symbolic Interactionist Perspective	Y	Y	Y	Y	Y	Y	Y	N	Y	Y	9/10	90%
Stubbs (2015)	Recruitment of nurses from India and their experiences of an Overseas Nurses Program	Y	Y	Y	Y	Y	Y	N	N	Y	Y	8/10	80%
Connor (2016)	Cultural Influence on Coping Strategies of Filipino Immigrant Nurses	Y	Y	U	Y	Y	Y	N	N	Y	Y	7/10	70%
Efendi et al. (2016)	Lived experience of Indonesian nurses in Japan: A phenomenological study	Y	Y	Y	Y	Y	Y	Y	Y	U	Y	9/10	90%
Healee (2016)	Working with difference: Thematic concepts of Japanese nurses working in New Zealand	Y	Y	Y	Y	Y	N	N	N	Y	Y	7/10	70%
Zhou et al. (2016)	Why do China-educated nurses emigrate? A qualitative exploration	Y	Y	Y	Y	Y	Y	Y	Y	Y	Y	10/10	100%
Stievano et al. (2017)	Indian nurses in Italy: a qualitative study of their professional and social integration	Y	Y	Y	Y	Y	Y	Y	Y	Y	Y	10/10	100%
Salami et al. (2018)	Downward occupational mobility of baccalaureate-prepared, internationally educated nurses to licensed practical nurses	Y	Y	Y	Y	Y	Y	Y	N	Y	Y	9/10	90%
Coustas (2019)	Factors impacting the retention of Registered Nurses recruited from India to work in South African hospitals: A case study	Y	Y	U	Y	Y	Y	N	N	Y	Y	7/10	70%
Nortvedt et al. (2019)	A courageous journey: Experiences of migrant Philippine nurses in Norway	Y	Y	Y	Y	Y	Y	Y	Y	Y	Y	10/10	100%
Philip et al. (2019)	Overseas Qualified Nurses’ (OQNs) perspectives and experiences of intraprofessional and nurse–patient communication through a Community of Practice lens	Y	Y	Y	Y	Y	Y	Y	Y	Y	Y	10/10	100%
Effendi et al. (2020)	The lived experience of Indonesian nurses in Kuwait: A phenomenological study	Y	Y	Y	Y	Y	Y	Y	Y	Y	Y	10/10	100%
Nursalam et al. (2020)	The Lived Experiences of Indonesian Nurses Who Worked as Care Workers in Taiwan	Y	Y	Y	Y	Y	N	N	N	Y	Y	7/10	70%
Almansour et al. (2021)	Home and expatriate nurses’ perceptions of job satisfaction: Qualitative findings	Y	Y	Y	Y	Y	Y	N	Y	Y	Y	9/10	90%
Dahl et al. (2021)	Motivation, Education, and Expectations: Experiences of Philippine Immigrant Nurses	Y	Y	Y	Y	Y	Y	Y	Y	Y	Y	10/10	100%
Yusuf et al. (2021)	Using social network tools to facilitate cultural adjustment of self- initiated Malaysian female expatriate nurses in Saudi Arabia	Y	Y	Y	U	Y	Y	N	Y	N	Y	7/10	70%
Effendi et al. (2022)	The course of broken dreams: The expectations and realities of the life of Indonesian nurses as care workers in Japan	Y	Y	Y	Y	Y	Y	Y	Y	Y	Y	10/10	100%
Joseph et al. (2022)	Transition Experiences of Indian Nurses Into Australian Mental Health System	Y	Y	Y	Y	Y	Y	Y	Y	Y	Y	10/10	100%

### Data extraction and synthesis

A data-based convergent synthesis was undertaken using the JBI approach to mixed-methods systematic reviews, during which the results of the included quantitative and mixed-methods studies were transformed into qualitative findings. Under this design, the data were analysed using the same synthesis method and combined and synthesised [28]. A three-step thematic synthesis proposed by Thomas and Harden [29] was performed. First, the texts were inductively coded; second, the resultant codes were compared and organised into categories to form ‘descriptive themes’; and, last, these themes were re-read and compared with evidence from the textual data of the included studies independently by two reviewers to generate ‘analytical themes’. Next, the themes were finalised after a consensus was reached between the two independent reviewers (DU and RP), with arbitration by a third reviewer (SYL) when required.

## Results

### Search outcomes

Our database search yielded 2003 records, of which we removed 759 duplicates. The remaining 1244 articles were subjected to title- and abstract screening, after which 1145 were deemed irrelevant. The full texts of the remaining 78 articles were assessed for eligibility, leading to the final inclusion of 27 studies (Fig. 1).

### Study characteristics

The 27 studies (19 qualitative and eight quantitative) comprised five each from Asia (Japan, Taiwan, Singapore and Malaysia), Australia and Europe (Italy, Norway and the United Kingdom); four each from the United States and the Middle East (Saudi Arabia and Kuwait); two from Canada; and one each from New Zealand and South Africa. The study characteristics and findings are outlined in Table 4. The studies collectively exhibited a medium-to-high methodological quality, as demonstrated by their overall ratings that ranged from 60 to 100%. Of note, our thematic synthesis yielded five themes—all of which were not country-specific and could be transferable between the geographical settings—as follows: (a) desire for better career prospects; (b) occupational downward mobility; (c) inequality in career advancement; (d) acculturation; and (e) support system.

**Table 4:** Characteristics of Included Studies

Authors, country	Study aim(s)	Study design	Sample characteristics	Key findings
Adhikari and Melia (2013), United Kingdom	To examine migrant nurses’ professional life in the UK	A multi-sited ethnographic approach using in-depth interviews	21 Nepal nurses	• Nepali nurses had aspirations to work in a technologically advanced and modern British hospital to further their career but eventually land in private sector nursing and care homes • Nepali nurses experienced downward professional mobility where valuable skills in acute-care nursing were gradually lost • Downward spiral of job dissatisfaction and lack of opportunity for career progression
Almansour et al. (2021), Saudi Arabia	To gain an understanding of the impact of expatriate status on nurses’ perceptions, by comparing the factors that influence job satisfaction among Saudi nurses to those that affect nurses recruited from other countries	A descriptive qualitative study using semi-structured interviews	8 Saudi Arabian nurses 6 Filipino nurses 4 Indian nurses 4 South African nurses 2 Jordanian nurses 2 Malaysian nurses	• Being away from family and friends significantly affected their happiness. Married contracts providing family accommodation and air tickets for family members were found to mitigate turnover intention • Language barriers as a fundamental factor influencing job satisfaction • The lack of opportunities for career advancement and further education had a negative influence on retention
Alshareef et al. (2020), Saudi Arabia	To identify and analyse the risk factors contributing to nursing turnover in Saudi Arabia and identifies practical solutions to decrease turnover and encourage nurses to stay in their jobs	A descriptive cross-sectional quantitative study using the 24-item Organizational Commitment Questionnaire	502 Asian nurses	• Organisational commitment is negatively correlated with anticipated turnover • Social support from immediate supervisor is negatively correlated with anticipated turnover • Autonomy also had a significant negative effect on anticipated turnover among nurses • Floating of staff – understaffed, heavy workload, pressure in working environment is negatively correlated with anticipated turnover
An et al. (2014), United States of America	To examine factors affecting the job satisfaction of immigrant Korean nurses	A descriptive cross-sectional quantitative study using a five-item Job Satisfaction Index (Brayfield and Rothe, 1951)	105 Korean nurses	• Job satisfaction showed a significantly negative correlation with perceived stress • Job satisfaction was significantly positively correlated with perceived organisational support • Job satisfaction was significantly positive correlated with self-efficacy
Connor (2016), United States	To understand culture’s influence on coping patterns and preferences among diverse populations	A cross-sectional qualitative descriptive design using semi-structured interviews	20 Filipino nurses	• The themes that emerged reflected similar coping behaviours and strategies and were categorised as (a) familial, (b) intracultural, (c) fate and faith-based, (d) forbearance (patience and self-control) and contentment, (e) affirming the nursing profession and proving themselves, and (f) escape and avoidance • Understanding the role of culture and adaptation on stress and coping behaviours is important to retain quality nurses and promote a healthier workplace
Coustas (2019), South Africa	To understand the obstacles and remediation required to retain the Registered Nurses recruited from India, and explore the hospital managements’ perceptions of these nurses’ contribution to their hospitals’ functioning	An instrumental case study	30 Indian nurses	• Deteriorating exchange rate negatively correlated with retention • Adjustment and the support of the spouse were critical to retention, expressed hopes that spouses would join them in South Africa • Role of management (workload, work environment) in retention of nurses from India
Dahl et al. (2021), Norway	To explore the educational experiences of Filipino nurses in the Philippines and expectations of their competence in Norway	An explorative design consisting using semi-structured interviews	10 Filipino nurses	• Nurses experienced a mismatch of expected competence, devalued by nurse credential process in Norway and experienced deskilling as an auxiliary nurse
Efendi et al. (2016), Japan	To develop a deeper understanding of the meaningful experiences of Indonesian nurses while working in Japanese hospitals	A phenomenological approach using semi-structured interviews	5 Indonesian nurses	• Six key themes were identified: (i) seeking better than before; (ii) communication challenges; (iii) the nursing examination as a culmination; (iv) differences in nursing practice; (v) cultural differences; and (vi) the benefits of living in developed country
Efendi et al. (2020), Kuwait	To explore the life experience of Indonesian nurses living and working in Kuwait	A phenomenological qualitative approach using semi-structured interviews	21 Indonesian nurses	• Similar culture and religious practices had a positive influence on retention • Linguistic incompetence an occupational stressor due to inability to engage in professional and social communication with confidence • Maintaining a close relationship with their family in the home country, increasing their mental strength, and engaging in social life to deal with homesickness
Efendi et al. (2022), Japan	To describe the narratives and experiences of Indonesian nurse migrants who worked as care workers in Japanese long-term care facilities	A descriptive qualitative study using semi-structured interviews	18 Indonesian nurses	• Nurses misunderstood the job scope and experienced deskilling due to a limited scope of practice • Access to religious infrastructure • Communication barrier despite a year of Japanese language training • A supportive leadership, peer support, emotional bonding with colleagues had a positive impact on retention
Geun et al. (2016), United States of America	To describe the gap between expected and perceived organisational experiences among Asian foreign-educated nurses (FENs) in the United States and to examine factors associated with turnover in their 1st year of employment	A descriptive cross-sectional quantitative study using Expectations and Experiences Measures as modified by Irving and Meyer	148 Korean nurses 44 Filipino nurses 4 Indian nurses 3 Taiwanese nurses 2 Chinese nurses	• Gap between expectations and experienced responsibilities is positively correlated with turnover intention • Significant differences between expectations and experiences in all 3 subscales: reward, responsibility, and comfort • Demographics and career characteristics is significantly associated with turnover intention
Geun et al. (2018), United States	To investigate factors affecting turnover of Asian foreign-educated nurses (FENs), which may lead to improvements in retention strategies	A descriptive cross-sectional quantitative study using the 24-item Organizational Commitment Questionnaire	148 Korean nurses 144 Filipino nurses 9 China, Indian and Taiwan nurses	• Perceived quality of orientation and affective commitment were significant predictors of turnover at the organisation level • perceived quality of orientation has important practical implications for human resources managers in nurse retention and the successful transition of Asian FENs
Goh & Lopez (2016), Singapore	To explore the job satisfaction level of migrant nurses working in a multicultural society and, more specifically, the relationship between their job satisfaction levels, work environment, their intentions to leave and the predictors of their intentions to leave	A descriptive correlational quantitative study using the 37-item Job Satisfaction Questionnaire	202 Filipino nurses 113 Malaysian nurses 81 Chinese nurses 45 Indian nurses 23 Myanmar nurses 31 Others	• A negative correlation between job satisfaction and each of the domains of the practice environment scale: participation in hospital affairs, nurse manager ability, staffing adequacy, nurse–physician relationship and nursing information technology • The ability of nurse managers to lead a ward and the practice environment are predictors of turnover intention
Healee (2016), New Zealand	To compare the differences experienced by Japanese nurses working in New Zealand from an organisational and personal perspective, using a qualitative approach	A descriptive qualitative study using semi-structured interviews	9 Japanese nurses	• An overall theme: finding a voice • Moving from a monoculture society with a traditional sense of duty, and a hierarchical approach to authority and working style to a society with a diverse culture, a flattened approach to authority, and a more active engagement in nursing • Ensuring that migrant nurses understand and adapt to a new socio-cultural and organisational environment, but also maintain their own cultural identity within it, has significant implications for the retention in the healthcare sector
Joseph et al. (2022), Australia	To explore the transition experiences of overseas-trained nurses from India currently working in mental health in Australia	A hermeneutic phenomenology approach using in-depth interviews	16 Indian nurses	• Difficulty acculturating due to strong Indian cultural beliefs • Loneliness in the absence of family, unfamiliarity with country and workplace
Lee et al. (2021), Malaysia	To examine the job satisfaction of the foreign-educated nurses in Malaysia, which includes the job satisfaction dimensions and the significant difference between sociodemographic status and job satisfaction	A descriptive cross-sectional quantitative study	29 Indian nurses 69 Filipino nurses 4 Pakistani nurses	• Job satisfaction was positively correlated with positive relationships with colleagues and superiors
Nortvedt et al. (2019), Norway	To explore how Philippine-educated nurses explain their choice of Norway as their migration destination and their experience with the credential assessment process in Norway	A hermeneutic design using qualitative research interviews	10 Filipino nurses	• The experienced were not getting jobs in the Norwegian healthcare system because they lacked credential recognition and sufficient Norwegian language skills • The fight for credential recognition—long and exhausting struggle with regulatory authorities
Nursalam et al. (2020), Taiwan	To elicit and describe the lived experiences of Indonesian nurses serving as care workers in Taiwan	A phenomenological qualitative approach using semi-structured interviews	16 Indonesian nurses	• Feelings of being trapped, victimised by fraud, losing their professional identity and skills • Difficult journey, communication inadequacy, limited career pathway • Feeling of being supported, support from management, support from recruiting agent may mitigate turnover intention
Philip et al. (2019), Australia	To explore the barriers and enablers of clinical communication experiences of OQNs from their perspective using a Communities of Practice framework	An exploratory qualitative study using semi-structured interviews	8 Filipino nurses 7 Indian nurses 1 Singaporean nurse 1 African nurse	• Lack of cultural orientation into work environment and this manifested in language causing uncertainties and confusion • Workplace interactions were unsatisfactory due to foreign accents, mispronunciation, or rapid speech • Adjustment for smooth transition: developing self-awareness, getting acquainted with Western cultural practices applicable to clinical setting via use of Australian colloquialisms
Primeau et al. (2021) Canada	To identify the main correlates of internationally educated nurses’ career satisfaction	A quantitative study using a self-developed questionnaire	86 Arabian nurses 880 Asian nurses 181 Black nurses 30 Latino nurses 774 White nurses	• Individual characteristics, namely age, gender, ethnicity (visible minority), parenting responsibility, and education, were all found to be significantly correlated to career satisfaction • Internationally educated nurses who experience discrimination are less satisfied with their nursing career
Salami et al. (2018), Canada	To explore the experience of baccalaureate-prepared, internationally educated nurses who work as licensed practical nurses in Canada	An exploratory transnational feminist qualitative study using semi-structured interviews	9 Filipino nurses 3 Indian nurses 1 Nigerian nurse 1 Mauritian nurse	• Migrating to Canada with hope for a better personal and professional life • A lack of knowledge and support about the RN registration process; difficulty in credential recognition and assessment; barriers in accessing bridging programmes; difficulty in passing language and RN registration examinations • Feeling dissatisfied due to a lack of training and leadership opportunities
Stievano et al. (2017), Italy	To investigate the lived subjective experiences of immigrant Indian nurses in Italy and specifically their professional and social integration	A descriptive qualitative study using semi-structured interviews	20 Indian nurses	• Experiencing difficult work situations and declining salary compared with higher incomes gained in more affluent countries (Australia, Canada, USA, UK) had a negative influence on retention • Lack of opportunity for career advancement, underemployed, organisational responsibilities, and restricted scope of practice had a negative influence on retention • Providing visas for families of nurses had a positive influence on retention
Stubbs (2015), United Kingdom	To explore the transition experiences of nurses recruited from India to London to work in critical care settings	A descriptive qualitative study using semi-structured interviews	16 Indian Nurses	• Differences in nurses’ role: autonomy and responsibility • Language: difficulty in understanding English accents • Pre-allocated mentors of similar culture aids their personal and professional integration into a new country
Yusuf et al. (2021), Saudi Arabia	To identify and explore the social network communication tools used to facilitate the adjustment process of Malaysian female expatriate nurses working in the Kingdom of Saudi Arabia (hereafter “the Kingdom” or “SA”) who are accompanied by neither their spouses nor families	A descriptive qualitative study using semi-structured interviews	16 Malaysian nurses	• Continuous engagement with family in country of origin may mitigate turnover intention • Social media communication tools being used by female expatriate nurses can help curb their loneliness and lessen the culture shock of living and working in a foreign country
Zanjani et al. (2021) Australia	To examine factors associated with OQNs’ sociocultural adjustment to the Australian healthcare system. A secondary aim was to determine whether there was a correlation between OQNs’ sociocultural adjustment and their mental and physical health	A descriptive cross-sectional quantitative study using 21-item revised version of the Sociocultural Adaptation Scale (SCAS-R)	84 Indian nurses 57 Filipino nurses 17 Chinese nurses 42 Others	• Sociocultural adaptation was positively associated with job satisfaction • Sociocultural adaptation was negatively correlated to perceived stress level • A supportive initial work environment, helpful responses to questions, robust orientation programme, respect for cultural differences, ability to demonstrate expertise, and effective communication channels were critical to successful long-term adaptation
Zhou (2014), Australia	To explore the ways in which China-educated nurses construct meaning regarding the experience of working in Australia	A constructivist grounded theory method using in-depth interviews	28 Chinese nurses	• Realising: difficult for participants to accept the washing, toileting, and feeding patients as there is moral obligation to look after sick family members in Chinese culture • Struggling: tension between the participants’ desire to hold on to their old selves and the need to conform to the new society • Reflecting: participants perceived that they were much unlikely to rise to managerial positions despite their relatively superior qualifications and greater seniority
Zhou et al. (2016), Australia	To explore factors influencing China-educated nurses to emigrate to Australia	A grounded theory approach using semi-structured interview	28 Chinese nurses	• (a) personal factors (to improve English, to see more of the world and cultures, to seek novelty and adventure); (b) work-related factors (better work environment and more career choices); (c) social factors (better living environment and lifestyle); (d) cultural factors (positive perceptions in China of those who emigrate or have overseas experiences), and (e) economic factors (higher salaries)

#### Theme 1: Desire for better career prospects

The most prevalent theme was Asian IENs’ search for better career prospects, on which 15 studies reported relevant findings. The factors that motivated Asian IENs to migrate were economic, work-related, and personal. As for the first factor, seven studies emphasised the role of economic factors, citing nurses’ desire to attain a better socio-economic position than that in their home country [30–36]. The specific reasons underlying the push factors for Asian IENs to leave their current host countries were the deteriorating exchange rate of the current host country [37], the unfair, discriminatory remuneration practices based on nationality [38], and the declining pay when compared with that in more affluent host countries [39].

Regarding work-related factors, three studies highlighted factors such as poor career paths, a lack of opportunities, a heavy workload and the poor public perception of nurses [34, 36, 37]; these factors also drove Asian IENs to search for better prospects in other countries. In addition, four studies reported that when Asian IENs’ professional autonomy was improved, they preferred continuing in their existing jobs [38–41]. In contrast, two studies found that organisational responsibilities negatively influenced their turnover intention [32, 42]. Last, personal factors, such as nurses’ desire to travel and experience different cultures and lifestyles, were identified in four studies [33, 39, 41, 43].

#### Theme 2: Occupational downward mobility

Occupational downward mobility undermined the retention of the Asian IENs, as highlighted in nine studies. Six studies reported that Asian IENs faced difficulties in credentialing assessments and recognition by local nursing regulatory bodies [30, 33, 34, 40, 44, 45]: the process was not only complex and time-consuming [30, 45] but also lacked transparency [34, 40]. Moreover, they experienced difficulty passing nursing licensure and language competence examinations [30, 34, 45]. Importantly, these nurses were subjected to occupational downgrading; since they were often relegated to a role lower than that of a registered nurse, pending completion of their recognition, this relegation resulted in their deskilling [30, 34, 40, 45]. In addition, education levels were a determinant of their career satisfaction, with diploma holders being the most satisfied, followed by bachelor-degree holders and higher-degree holders in that order [46].

As reported in eight studies, disparities between the Asian IENs’ expectations and reality represented another critical aspect. Five studies highlighted that differences in nursing practices could influence their retention. For example, in developing countries such as China, Indonesia, and the Philippines, patients' families often perform basic nursing care (e.g. feeding, personal cleanliness, dressing, and elimination). Conversely, in developed countries, Asian IENs would be expected to undertake such activities for patients [30, 33, 39, 41, 42]. Moreover, the remaining three studies reported a similar disparity for Asian IENs employed in either long-term care facilities (where the scope of practice might be limited) or other incongruous settings (where their previous experiences or expertise might be irrelevant), with resultant deskilling among them [37, 42, 47]. Similarly, Primeau et al. [47] have corroborated that Asian IENs in long-term care facilities exhibited lower career satisfaction than their hospital-based counterparts. Such disparities have led to frustration and disempowerment among Asian IENs and compromised their retention [32, 37].

#### Theme 3: Inequality in career advancement

Seven studies suggested that inequality in opportunities for career advancement compromises Asian IENs’ retention. Four studies identified a lack of access to advanced education [33, 34, 48, 49]. The remaining three reported a lack of organisational or leadership responsibilities [34, 37, 48] as a cause of limited career progression. Furthermore, Nursalam et al. [33] highlighted unclear career pathways. The Asian IENs also reported that they were given fewer opportunities for career development than host-country nurses [38, 46]. Last, an unfair performance appraisal system and a nationality-based remunerative system were found to be predictors of turnover [38].

#### Theme 4: Acculturation

Acculturation and its various aspects critically influenced the Asian IENs’ retention, as demonstrated in 13 studies. Sociocultural adaptation correlated positively with the Asian IENs’ career satisfaction [35] and retention [50], but ten studies highlighted that communication barriers hindered their acculturation. The challenges they faced were manifold. For instance, they needed to learn a new language [33, 42, 45]; their communication skills were inadequate [40, 51]; they found it challenging to understand informal language [39, 41]; they were unfamiliar with local accents and were unable to cope with the communication speed of those who used the local language [41, 51]; and they were unfamiliar with dialects [35, 41]. Furthermore, four studies underlined the role of English proficiency in affecting career satisfaction [35, 44, 51, 52].

As with languages, cultural identity is socially constructed. Difficulties in acculturation due to deep-rooted cultural beliefs upheld by Chinese [41], Indian [40, 53] and Japanese migrant nurses [43] have been documented. Moreover, cultural distance—a collective term denoting differences in religious beliefs, ethnicity, social norms, and languages—predicts the Asian IENs’ retention, as shown in four studies [31, 35, 44, 52]. Nonetheless, some encouraging evidence has suggested that Asian IENs employed in Asian countries could acculturate more rapidly than those employed in non-Asian countries [31, 35, 44, 52].

#### Theme 5: Support system

The lack of a support system for Asian IENs and the concomitant isolation from family support structures/systems were identified in seven studies to correlate negatively with their retention. In the physical absence of their family [40, 45, 49, 53], the Asian IENs’ continuous remote engagement with their family promoted their emotional stability, thereby contributing to retention [42, 54]. Similarly, providing visas or contracts to their spouse and children positively influenced their retention [37, 38, 48]. Moreover, Almansour et al. [49] reported that Asian IENs in Saudi Arabia with ‘married-status’ contracts, which provide accommodation, hospitalisation coverage and air tickets for their families, had higher satisfaction levels than their counterparts with ‘single-status’ contracts.

Further, seven studies suggested that social support from an immediate supervisor favoured the Asian IENs’ retention [40, 45, 49, 53]. Using the Anticipated Turnover Scale, Alshareef et al. [38] found such support to be a significant predictor of anticipated turnover. Thus, creating a supportive work environment is crucial since wholesome collegial interactions likely reduce workplace stress and promote physical and psychological well-being [42, 44]. Notably, perceived stress impaired not only sociocultural adaptation [35], but also job satisfaction [50], resulting in calls for stress-management programmes [50].

## Discussion

Our synthesis of 27 articles through a systematic review uncovers insightful existing knowledge about the underlying factors influencing Asian IENs’ retention in their host countries, in line with which the relevant authorities can devise retention strategies. Our thematic analysis demonstrated that, across the included articles, the Asian IENs’ search for better career prospects emerged as the most prevalent theme. Asian IENs, motivated to attain a higher social and economic standing, migrate with high expectations in pursuit of enhanced remuneration to improve their socio-economic position and provide a better future for their families via remittances [32]. While economic factors may predominate in the Asian IENs’ consideration, these are not the sole impetus. Work-related factors, such as more attractive opportunities for career advancement and further education, also drive them to migrate out of their current host countries. In addition, the Asian IENs anticipate a favourable working environment that would provide them with societal respect, a low workload, autonomy and better career paths [34]. Personal factors, such as the search for a better quality of life, have also spurred nurses from China, Japan and Singapore to explore different lifestyles abroad [41, 43].

Overall, the common denominator across all such factors is the Asian IENs' constant desire to seek destinations with better opportunities, given the ease of international mobility [55]. For example, in the Philippines–Singapore ‘bus-stop’ migration model, Filipino nurses build their skill sets and experiences in Singapore and then attempt to move to other countries that more readily grant permanent residency [12]. Accordingly, the implication is that policymakers need to acknowledge the phenomenon of such mobility and provide direct pathways to secure long-term settlement and retention of Asian IENs in host countries, such as offering them permanent residency and professional advancement.

Moreover, occupational downward mobility is another theme that contributes to the Asian IENs’ turnover. During their transition, they face challenges that lead to their deskilling: delays in assessments and recognition of their nursing credentials [34], devaluation of their previous nursing qualifications during accreditation [44], difficulty in passing nursing licensure and language-competence examinations [45]; and relegation, either to a role with a job scope inferior to that of a registered nurse or to areas not matching their previous experiences [47]. Consequently, this finding calls for greater transparency in the credential requirements for acquiring a nursing practice licence in host countries and the accessibility towards recognising nursing credentials from the country of origin (e.g. the duration of application) [40]. To promote transparency in accreditation across borders, universal referential systems, such as the European Qualifications Framework, which relates different countries' national qualifications systems to a common European reference standard, can be adopted [56].

Another aspect under this theme is the disparities between the Asian IENs’ expectations and reality. Although they possess credentials similar to that of local nurses [34], most Asian IENs are placed in settings not commensurate with their previous education or expertise, resulting in their deskilling [30]. Asian IENs with higher educational qualifications exhibited lower job satisfaction [46]. This finding highlights the need for flexible bridging programmes initiated at the organisational level to match their competencies and qualifications to their new places of practice [34]. Other mismatches are also notable. For instance, Asian IENs recruited into long-term care facilities may have a limited scope of practice [57] since they originate from countries where nursing homes and care homes might be less prevalent. Thus, they may find themselves entering employment with a warped understanding of the job scope [42, 47]. Furthermore, in countries such as China, Indonesia and the Philippines, families often deliver basic nursing care to patients in the wards of tertiary hospitals [30, 41, 42]; however, in some Asian IENs’ host countries, the nursing staff is expected to provide such care. Such discordance between job expectations and existing care delivery practices may contribute to feelings of devaluation and disempowerment among Asian IENs. Accordingly, organisations need to adopt recruitment approaches that detail the nursing job scope in the prospective applicants’ native language so that they can make informed decisions before migrating [33, 37, 47].

Inequality in career progression, the third theme evident in this review, represents an unspoken perception among Asian IENs. Compared with host-country nurses, Asian IENs reported fewer opportunities for career advancement, fewer opportunities for further education, a lack of organisational responsibilities and a restricted scope of practice due to deskilling [37, 49]. In this context, it is critical to acknowledge the importance of professional development as a principal pull factor for migration by Asian IENs. To address this implicit but critical perception, organisations should be transparent with Asian IENs' prospects of career progression at the early stage of their career [37, 42, 49].

Acculturation—the fourth theme we identified in this review—is the psychological adjustment Asian IENs undergo on migrating across geographical borders. Significantly, individuals' interpretation of a social environment provides an orderly, common-sense process of which they are unaware [58]. This process is shaped by the individuals' language and ethnicity and how they maintain their culture in public and private settings [50]. Within this context, communication styles are influenced by communication speeds, informal language, accents, intonation, dialects, and colloquial terms established through longstanding usage. Hence, these communication styles are intricately nuanced and highly contextual [39, 40]. As language is a social construct, and by providing advanced language training can improve Asian IENs' intercultural communication [39, 51] and facilitate the ease of acculturation [50].

We observed that nurses who migrate to countries with a shorter cultural distance demonstrate accelerated cultural adaptation, such as Asian IENs who move to another Asian country [31, 52]. This finding highlights the need for healthcare organisations to consider cultural distance before recruitment. Multiple studies have emphasised the importance of a robust orientation programme for the Asian IENs’ smooth integration into the host country’s social system [32, 48, 54, 59]. Corroborative evidence has also been provided by Redman et al. [60], who reported a negative correlation between nurses’ perceived quality of orientation efforts and turnover intention. In this context, orientation regarding culture and social norms should be bilateral to facilitate mutual respect and empathy for cultural differences [35]. In addition to cultural orientation, studies have identified the need for an induction programme for successful long-term adaptation [32, 40, 44, 48, 60], under which strategies include pairing the Asian IENs with local nurses [44] or with host nurses from similar cultures [40].

A robust support system—the fifth theme that our review revealed—has been identified as a crucial determinant in acclimatising Asian IENs. Amidst struggles with loneliness and homesickness [49, 53], Asian IENs must build a social network from scratch and familiarise themselves with their host country's local social activities and norms [39, 45]. Within this context, their families play a part in mitigating their isolation, and this familial presence contributes to their sustained retention [61]. Notably, married Asian IENs with children were more satisfied than those without [46, 49], which might be attributed to the positive association between family-life satisfaction and job satisfaction [62]. Thus, given the mitigating role of the Asian IENs’ families, the implication of this finding at the national level is to give Asian IENs married-status contracts and issue visas to their next of kin, contributing to the sustained retention of Asian IENs.

Likewise, a social network independent from families is important to curb isolation. Upon settling into a culturally different environment, the Asian IENs must rebuild a local social network [39, 45]. Hence, organisations should introduce regular social gatherings involving all staff to promote a sense of belonging among the Asian IENs [35, 39, 41]. Through undergoing and sharing everyday experiences, relatable conversational topics can be initiated to accelerate acculturation and mitigate isolation [45]. This process can be further enhanced if the Asian IENs integrate into the local community by aligning themselves with similar cultures and languages [37, 44].

Last, supportive supervision, strong leadership from immediate supervisors, and interpersonal relationships are critical in promoting a positive work environment for Asian IENs [38]. In addition, healthcare institutions should implement culturally appropriate stress-management programmes as part of an organisational health promotion strategy [59]. In particular, acculturation extends beyond the initial orientation stage. A comprehensive support system implemented from the individual to the organisational level will enable successful long-term adaptation by Asian IENs [35]. Following the easing of onboarding stressors, such as language barriers, alienation, and culture shock, the Asian IENs are envisioned to establish better communication skills and build their social networks, thereby contributing to their sustained retention.

### Strengths and limitations

This review has included studies across settings with different cultural and social constructs, encompassing not only Asian and non-Asian host countries, but also developed and developing countries. This strength provided a differentiated examination of Asian IENs across the various themes. Nonetheless, some significant limitations remain. For example, some included studies focused solely on career satisfaction instead of turnover intention. However, career satisfaction and turnover intention are likely correlated [63]. Further, the reviewed studies did not explore migratory patterns and their associated implications post-COVID-19. In addition, grey literature and non-English publications were excluded from this review.

### Implications for administrators

This review offers an analysis of the factors influencing the retention of Asian IENs in host countries, which healthcare organisations can use as a basis to develop staff retention strategies and evaluate their turnover rates. First, given the Asian IENs’ international mobility and migratory options, host-country healthcare organisations should ensure that these individuals’ intrinsic and extrinsic motivations are met, especially regarding granting permanent residency. Second, healthcare organisations should comprehensively detail the nursing practice environment and job scope during their recruitment efforts, while nursing regulatory bodies should ensure transparency in credentialing assessment and recognition to avoid mismatches between reality and Asian IENs’ expectations and, thus, prevent their disillusionment. Third, healthcare organisations should ensure equality and transparency in communicating career advancement prospects and further education opportunities. Fourth, with due consideration of cultural distance, healthcare organisations should institute differentiated orientation and induction programmes that are more sensitive to the cultural needs of the Asian IENs to help them adapt more effectively to the new environment. Next, robust support systems should be in place to facilitate Asian IENs’ social integration, during which their new local social network and family support are critical. To further promote their sustained retention in host countries, policymakers may also consider providing visas and contracts to their families. Finally, healthcare organisations should cultivate a wholesome working environment by upholding supportive supervision and solid collegial relationships.

## Conclusion

In this systematic review, we have thematically explored the factors influencing the retention of Asian IENs in host countries. Studies on their motivations and expectations have revealed that Asian IENs' constant search for better career prospects drives them to migrate. However, upon employment in the host countries, they face occupational downgrading in the profession, leading to their deskilling and mismatched expectations; moreover, they experience inequality in career progression. They also face further challenges in the acculturation stage; it has been found that during this stage, a robust support system and positive interpersonal relationships expedite their sociocultural adaptation. Our review has also identified potential strategies for policymakers to retain Asian IENs. Although increasing the number of recruitments drives to address nursing shortages may represent a quick solution, policymakers should avoid such a myopic outlook: they should address the root causes of high turnover and re-evaluate the efficaciousness and sustainability of their existing staff retention measures. Future research may explore downstream factors influencing Asian IENs’ retention, such as adverse working conditions, burnout or challenges faced by specific vulnerable groups, to provide more targeted solutions to issues concerning the retention of Asian IENs.

### Supplementary Information

**Additional file 1.** Index terms and keywords for searching.

## Data Availability

Data will be made available through contacting the corresponding author.
