# Supplementary material for: Global migration and factors influencing retention of Asian internationally educated nurses: a systematic review
Source: Hum Resour Health. 2024 Mar 1;22:17. doi: 10.1186/s12960-024-00900-5 (PMC10905872; doi:10.1186/s12960-024-00900-5)
Supplement: Supplementary file 1 — Additional file 1. Index terms and keywords for searching. [file 12960_2024_900_MOESM1_ESM.docx]

**Additional File 1: Index Terms and Keywords for Searching**

| **Database** | **Indexed terms and keywords** |
| --- | --- |
| **PubMed** | #1 International[Title/Abstract] OR overseas[Title/Abstract] OR migra*[Title/Abstract]  #2 nurs*[Title/Abstract]  #3 #1 AND #2  #4 "overseas trained nurse*"[Title/Abstract] OR "overseas nurse*"[Title/Abstract] OR "overseas qualified nurse*"[Title/Abstract] OR "overseas qualified nurse*"[Title/Abstract] OR "internationally recruited nurse*"[Title/Abstract] OR "internationally educated nurse*"[Title/Abstract] OR "internationally qualified nurse*"[Title/Abstract] OR "international nurse*"[Title/Abstract] OR "immigrant nurse*"[Title/Abstract] OR "foreign nurse*"[Title/Abstract] OR "foreign trained nurse*"[Title/Abstract] OR "foreign educated nurse*"[Title/Abstract] OR "migrant nurse*"[Title/Abstract]  #5 #3 OR #4  #6 Retain*[Title/Abstract] OR Turnover[Title/Abstract] OR Attrition[Title/Abstract] OR Retention[Title/Abstract] OR Loyalty[Title/Abstract] OR Intention to quit[Title/Abstract] OR Intention to stay[Title/Abstract] OR Leav*[Title/Abstract] OR Quit[Title/Abstract]  #7 Experience[Title/Abstract] OR Perceptions[Title/Abstract] OR Attitudes[Title/Abstract] OR Views[Title/Abstract] OR Feelings[Title/Abstract] OR Challenges[Title/Abstract]  #8 “Personnel Turnover”[MeSH]  #9 #6 OR #7 OR #8  #10 Asia [Title/Abstract] OR Borneo[Title/Abstract] OR Brunei[Title/Abstract] OR Cambodia[Title/Abstract] OR China[Title/Abstract] OR "Hong Kong"[Title/Abstract] OR India[Title/Abstract] OR Indonesia[Title/Abstract] OR Japan[Title/Abstract] OR Korea[Title/Abstract] OR Laos[Title/Abstract] OR Myanmar[Title/Abstract] OR Malaysia[Title/Abstract] OR Philippines[Title/Abstract] OR Singapore[Title/Abstract] OR Taiwan*[Title/Abstract] OR Thailand[Title/Abstract] OR Vietnam[Title/Abstract]  #11 Asian[Title/Abstract] OR Burmese[Title/Abstract] OR Cambodian[Title/Abstract] OR Chinese[Title/Abstract] OR Indian[Title/Abstract] OR Indonesian[Title/Abstract] OR Japanese[Title/Abstract] OR Korean[Title/Abstract] OR Malaysian[Title/Abstract] OR Filipino[Title/Abstract] OR Singaporean[Title/Abstract] OR Thai[Title/Abstract] OR Taiwanese[Title/Abstract] OR Vietnamese[Title/Abstract] OR  Asia [Title/Abstract] OR Borneo[Title/Abstract] OR Brunei[Title/Abstract] OR Cambodia[Title/Abstract] OR China[Title/Abstract] OR "Hong Kong"[Title/Abstract] OR India[Title/Abstract] OR Indonesia[Title/Abstract] OR Japan[Title/Abstract] OR Korea[Title/Abstract] OR Laos[Title/Abstract] OR Myanmar[Title/Abstract] OR Malaysia[Title/Abstract] OR Philippines[Title/Abstract] OR Singapore[Title/Abstract] OR Taiwan*[Title/Abstract] OR Thailand[Title/Abstract] OR Vietnam[Title/Abstract] OR Asian[Title/Abstract] OR Burmese[Title/Abstract] OR Cambodian[Title/Abstract] OR Chinese[Title/Abstract] OR Indian[Title/Abstract] OR Indonesian[Title/Abstract] OR Japanese[Title/Abstract] OR Korean[Title/Abstract] OR Malaysian[Title/Abstract] OR Filipino[Title/Abstract] OR Singaporean[Title/Abstract] OR Thai[Title/Abstract] OR Taiwanese[Title/Abstract] OR Vietnamese[Title/Abstract] Asia [Title/Abstract] OR Borneo[Title/Abstract] OR Brunei[Title/Abstract] OR Cambodia[Title/Abstract] OR China[Title/Abstract] OR "Hong Kong"[Title/Abstract] OR India[Title/Abstract] OR Indonesia[Title/Abstract] OR Japan[Title/Abstract] OR Korea[Title/Abstract] OR Laos[Title/Abstract] OR Myanmar[Title/Abstract] OR Malaysia[Title/Abstract] OR Philippines[Title/Abstract] OR Singapore[Title/Abstract] OR Taiwan*[Title/Abstract] OR Thailand[Title/Abstract] OR Vietnam[Title/Abstract] OR Asian[Title/Abstract] OR Burmese[Title/Abstract] OR Cambodian[Title/Abstract] OR Chinese[Title/Abstract] OR Indian[Title/Abstract] OR Indonesian[Title/Abstract] OR Japanese[Title/Abstract] OR Korean[Title/Abstract] OR Malaysian[Title/Abstract] OR Filipino[Title/Abstract] OR Singaporean[Title/Abstract] OR Thai[Title/Abstract] OR Taiwanese[Title/Abstract] OR Vietnamese[Title/Abstract]  **Search outcome: 378 results** |
| **EMBASE** | #1 'international':ab,ti OR 'overseas':ab,ti OR 'migra*':ab,ti  #2 'nurse'/exp  #3 #1 AND #2  #4 'overseas trained nurse*':ab,ti OR 'overseas nurse*':ab,ti OR 'overseas-qualified nurse*':ab,ti OR 'overseas qualified nurse*':ab,ti OR 'internationally recruited nurse*':ab,ti OR 'internationally educated nurse*':ab,ti OR 'internationally qualified nurse*':ab,ti OR 'international nurse*':ab,ti OR 'immigrant nurse*':ab,ti OR 'foreign nurse*':ab,ti OR 'foreign trained nurse*':ab,ti OR 'foreign educated nurse*':ab,ti OR 'migrant nurse*':ab,ti  #5 #3 OR #4  #6 retain*:ti,ab,kw OR turnover:ti,ab,kw OR attrition:ti,ab,kw OR retention:ti,ab,kw OR loyalty:ti,ab,kw OR 'intention to quit':ti,ab,kw OR 'intention to stay':ti,ab,kw OR leav*:ti,ab,kw OR quit:ti,ab,kw OR experience:ti,ab,kw OR perceptions:ti,ab,kw OR attitudes:ti,ab,kw OR views:ti,ab,kw OR feelings:ti,ab,kw OR challenges:ti,ab,kw  #7 asia:ti,ab,kw OR borneo:ti,ab,kw OR brunei:ti,ab,kw OR cambodia:ti,ab,kw OR china:ti,ab,kw OR 'hong kong':ti,ab,kw OR india:ti,ab,kw OR indonesia:ti,ab,kw OR japan:ti,ab,kw OR korea:ti,ab,kw OR laos:ti,ab,kw OR myanmar:ti,ab,kw OR malaysia:ti,ab,kw OR philippines:ti,ab,kw OR singapore:ti,ab,kw OR taiwan*:ti,ab,kw OR thailand:ti,ab,kw OR vietnam:ti,ab,kw OR asian:ti,ab,kw OR burmese:ti,ab,kw OR cambodian:ti,ab,kw OR chinese:ti,ab,kw OR indian:ti,ab,kw OR indonesian:ti,ab,kw OR japanese:ti,ab,kw OR korean:ti,ab,kw OR malaysian:ti,ab,kw OR filipino:ti,ab,kw OR singaporean:ti,ab,kw OR thai:ti,ab,kw OR taiwanese:ti,ab,kw OR vietnamese:ti,ab,kw  #8 #5 AND #6 AND #7  **Search outcome: 126 results** |
| **CINAHL** | S1 TI ( International* OR overseas OR migra* OR Foreign* ) OR AB ( International* OR overseas OR migra* OR Foreign* )  S2 TI Nurs* OR AB Nurs*  S3 S1 and S2  S4 TI ( “overseas trained nurs*” OR “overseas nurs*” OR “overseas-qualified nurs*” OR “overseas qualified nurs*” OR “overseas educated nurs*” OR “internationally recruited nurs*” OR “internationally educated nurs*” OR “internationally qualified nurs*” OR “international nurs*” OR “immigrant nurs*” OR “foreign nurs*” OR “foreign trained nurs*” OR “foreign educated nurs*” OR “migrant nurs*” ) OR AB ( “overseas trained nurs*” OR “overseas nurs*” OR “overseas-qualified nurs*” OR “overseas qualified nurs*” OR “overseas educated nurs*” OR “internationally recruited nurs*” OR “internationally educated nurs*” OR “internationally qualified nurs*” OR “international nurs*” OR “immigrant nurs*” OR “foreign nurs*” OR “foreign trained nurs*” OR “foreign educated nurs*” OR “migrant nurs*” )  S5 S3 OR S4  S6 TI ( retain* OR turnover OR attrition OR retention OR loyalty OR intention to quit OR intention to stay OR leav* OR quit OR ) OR AB ( retain* OR turnover OR attrition OR retention OR loyalty OR intention to quit OR intention to stay OR leav* OR quit)  S7 TI ( Experience OR perceptions OR attitudes OR views OR feelings OR challenges ) OR AB ( Experience OR perceptions OR attitudes OR views OR feelings OR challenges )  S8 MH Personnel Turnover  S9 S6 OR S7 OR S8  S10 TI ( Asia OR Borneo OR Brunei OR Cambodia OR China OR "Hong Kong" OR India OR Indonesia OR Japan OR Korea OR Laos OR Myanmar OR Malaysia OR Philippines OR Singapore OR Taiwan* OR Thailand OR Vietnam OR Asian OR Burmese OR Cambodian OR Chinese OR Indian OR Indonesian OR Japanese OR Korean OR Malaysian OR Filipino OR Singaporean OR Thai OR Taiwanese OR Vietnamese ) AND AB ( Asia OR Borneo OR Brunei OR Cambodia OR China OR "Hong Kong" OR India OR Indonesia OR Japan OR Korea OR Laos OR Myanmar OR Malaysia OR Philippines OR Singapore OR Taiwan* OR Thailand OR Vietnam OR Asian OR Burmese OR Cambodian OR Chinese OR Indian OR Indonesian OR Japanese OR Korean OR Malaysian OR Filipino OR Singaporean OR Thai OR Taiwanese OR Vietnamese )  S11 S5 AND S9 AND S10  Filters applied: Language: English; Source Type: Academic journals  **Search outcome: 171 results** |
| **Scopus** | ( ( ( TITLE-ABS-KEY ( international OR overseas OR migra* ) AND TITLE-ABS-KEY ( nurs* ) ) ) OR ( TITLE-ABS-KEY ( "overseas trained nurs*" OR "overseas nurs*" OR "overseas-qualified nurs*" OR "overseas qualified nurs*" OR "overseas educated nurs*" OR "internationally recruited nurs*" OR "internationally educated nurs*" OR "internationally qualified nurs*" OR "international nurs*" OR "immigrant nurs*" OR "foreign nurs*" OR "foreign trained nurs*" OR "foreign educated nurs*" OR "migrant nurs*" ) ) ) AND ( TITLE-ABS-KEY ( retain* OR turnover OR attrition OR retention OR loyalty OR "intention to quit" OR “intention to stay” OR leav* OR quit OR experience OR 'perceptions OR attitudes' OR views OR feelings OR challenges ) ) AND ( TITLE-ABS-KEY ( asia OR borneo OR brunei OR cambodia OR china OR "hong kong" OR india OR indonesia OR japan OR korea OR laos OR myanmar OR malaysia OR philippines OR singapore OR taiwan OR thailand OR vietnam OR asian OR burmese OR cambodian OR chinese OR indian OR indonesian OR japanese OR korean OR malaysian OR filipino OR singaporean OR thai OR taiwanese OR vietnamese ) )  Filters applied: Source Type: Journal articles; Language: English  **Search outcome: 771 results** |
| **Web of Science** | **#**1 (TI=(International OR overseas OR migra*)) OR AB=(International OR overseas OR migra*)  #2 (TI=(nurs*)) OR AB=(nurs*)  #3 #1 AND #2  #4 (TI=("overseas trained nurs*" OR "overseas nurs*" OR "overseas-qualified nurs*" OR "overseas qualified nurs*" OR "overseas educated nurs*" OR "internationally recruited nurs*" OR "internationally educated nurs*" OR "internationally qualified nurs*" OR "international nurs*" OR "immigrant nurs*" OR "foreign nurs*" OR "foreign trained nurs*" OR "foreign educated nurs*" OR "migrant nurs*" )) OR AB=("overseas trained nurs*" OR "overseas nurs*" OR "overseas-qualified nurs*" OR "overseas qualified nurs*" OR "overseas educated nurs*" OR "internationally recruited nurs*" OR "internationally educated nurs*" OR "internationally qualified nurs*" OR "international nurs*" OR "immigrant nurs*" OR "foreign nurs*" OR "foreign trained nurs*" OR "foreign educated nurs*" OR "migrant nurs*" )  #5 #3 OR #4  #6 (TI=(retain* OR turnover OR attrition OR retention OR loyalty OR “intention to quit” OR “intention to stay” OR leav* OR quit OR experience OR 'perceptions OR attitudes' OR views OR feelings OR challenges)) OR AB=(retain* OR turnover OR attrition OR retention OR loyalty OR “intention to quit” OR “intention to stay” OR leav* OR quit OR experience OR 'perceptions OR attitudes' OR views OR feelings OR challenges)  #7 (TI=(Asia OR Borneo OR Brunei OR Cambodia OR China OR "Hong Kong" OR India OR Indonesia OR Japan OR Korea OR Laos OR Myanmar OR Malaysia OR Philippines OR Singapore OR Taiwan* OR Thailand OR Vietnam OR Asian OR Burmese OR Cambodian OR Chinese OR Indian OR Indonesian OR Japanese OR Korean OR Malaysian OR Filipino OR Singaporean OR Thai OR Taiwanese OR Vietnamese )) OR AB=(Asia OR Borneo OR Brunei OR Cambodia OR China OR "Hong Kong" OR India OR Indonesia OR Japan OR Korea OR Laos OR Myanmar OR Malaysia OR Philippines OR Singapore OR Taiwan* OR Thailand OR Vietnam OR Asian OR Burmese OR Cambodian OR Chinese OR Indian OR Indonesian OR Japanese OR Korean OR Malaysian OR Filipino OR Singaporean OR Thai OR Taiwanese OR Vietnamese )  #8 #5 AND #6 AND #7  **Search outcome: 446 results** |
| **PsycINFO** | #1 (International or overseas or migra*).ti. or (International or overseas or migra*).ab.  #2 nurs*.ti. or nurs*.ab.  #3 #1 and #2  #4 ("overseas trained nurs*" or "overseas nurs*" or "overseas-qualified nurs*" or "overseas qualified nurs*" or "overseas educated nurs*" or "internationally recruited nurs*" or "internationally educated nurs*" or "internationally qualified nurs*" or "international nurs*" or "immigrant nurs*" or "foreign nurs*" or "foreign trained nurs*" or "foreign educated nurs*" or "migrant nurs*").ti. or ("overseas trained nurs*" or "overseas nurs*" or "overseas-qualified nurs*" or "overseas qualified nurs*" or "overseas educated nurs*" or "internationally recruited nurs*" or "internationally educated nurs*" or "internationally qualified nurs*" or "international nurs*" or "immigrant nurs*" or "foreign nurs*" or "foreign trained nurs*" or "foreign educated nurs*" or "migrant nurs*").ab.  #5 #3 or #4  #6 (retain* or turnover or attrition or retention or loyalty or intention to quit or intention to stay or leav* or quit or experience or perceptions or attitudes or views or feelings or challenges).ti. or (retain* or turnover or attrition or retention or loyalty or intention to quit or intention to stay or leav* or quit or experience or perceptions or attitudes or views or feelings or challenges).ab.  #7 (asia or borneo or brunei or cambodia or china or "hong kong" or india or indonesia or japan or korea or laos or myanmar or malaysia or philippines or singapore or taiwan or thailand or vietnam or asian or burmese or cambodian or chinese or indian or indonesian or japanese or korean or malaysian or filipino or singaporean or thai or taiwanese or vietnamese).ti. or (asia or borneo or brunei or cambodia or china or "hong kong" or india or indonesia or japan or korea or laos or myanmar or malaysia or philippines or singapore or taiwan or thailand or vietnam or asian or burmese or cambodian or chinese or indian or indonesian or japanese or korean or malaysian or filipino or singaporean or thai or taiwanese or vietnamese).ab.  #8 #5 and #6 and #7  **Search outcome: 111 results** |
